# Utilization of Quercetin as an Oviposition Stimulant by Lab-Cultured *Coleomegilla* *maculata* in the Presence of Conspecifics and a Tissue Substrate

**DOI:** 10.3390/insects9030077

**Published:** 2018-06-29

**Authors:** Eric W. Riddick, Zhixin Wu, Fred J. Eller, Mark A. Berhow

**Affiliations:** 1 National Biological Control Laboratory, USDA-ARS, Stoneville, MS 38776, USA; zhixin.wu@ars.usda.gov; 2 Functional Foods Research Unit, USDA-ARS, Peoria, IL 61604, USA; fred.eller@ars.usda.gov (F.J.E.); mark.berhow@ars.usda.gov (M.A.B.)

**Keywords:** aphids, biological control, bioflavonoids, Coccinellidae, lady beetle, predator, rearing, reproduction

## Abstract

Background: The discovery of natural products to improve the reproductive performance of mass-reared predators is an important aim for successful augmentative biological control. We tested the hypothesis that quercetin (a bioflavonoid) stimulates oviposition by the ladybird beetle *Coleomegilla* *maculata* in the presence of conspecifics and a tissue substrate. Methods: We conducted bioassays in solitary cages (housing one female) and communal cages (housing 10 females) to estimate daily oviposition site preferences, egg production in response to quercetin in the presence or absence of a tissue paper substrate, and female “resting” positions. Results: Females preferentially oviposited within 1–2 cm of quercetin powder, held in a tiny dish, at the base of cages. When given a choice, females oviposited in the dish with quercetin over a tissue paper substrate. In one of two experiments, they produced more egg clutches, regardless of oviposition site, when the quercetin and tissue were in close juxtaposition. Females “rested” on the tissue in the presence or absence of quercetin. Conclusion: This study provides evidence that quercetin can be utilized as an oviposition stimulant by *C*. *maculata* in a rearing system. Future research should determine if quercetin stimulates oviposition in other ladybird beetle species.

## 1. Introduction

A major impediment to mass-producing insect predators in quantities that are necessary for augmentative biological control is encouraging females to oviposit their full potential of eggs when reared on non-prey foods [1,2]. In addition, chemical cues that guide females to suitable oviposition sites are often connected to natural prey [3,4]. In artificial rearing systems, many of these cues are completely lacking. Consequently, females decide to withhold some of their eggs or choose to lay them on other substrates, often of synthetic origin. Some commonly used substrates are tissue paper, filter paper, and aluminum sheets [5,6,7]. In the absence of any oviposition substrates, females might opt to lay their eggs on the underside of the lid or sidewalls of the rearing container, vial, or Petri dish [8,9].

Very few plant-derived natural products are known to stimulate the oviposition in predators including ladybirds. An extract that was prepared from the leaves and wood of European barberry (*Berberis vulgaris* L.) purportedly stimulated oviposition in the ladybird beetles, *Adalia bipunctata* (L.) and *Coccinella septempunctata* L., when sprayed on the leaves of sour cherry (*Prunus cerasus* L.), which is not a preferred plant for oviposition [10]. Wood panels, wood extracts, and several fractions from eastern redcedar (*Juniperus virginiana* L.) stimulated oviposition in four ladybirds, *A*. *bipunctata*, *Coccinella transversoguttata* Faldermann, *Cycloneda munda* (Say), and *Coleomegilla maculata* (DeGeer) [11,12]. Polyphenols in redcedar wood were thought to be the source of the compounds stimulating oviposition [11,12]. In our recent study, several fractions (containing polyphenols) that were isolated from redcedar heartwood stimulated oviposition by *C*. *maculata* in cage bioassays; some of the active fractions were bioflavonoids, such as taxifolin, quercetin, and naringenin (to a lesser extent) [13]. Additional bioassays using synthetic compounds confirmed the strong, stimulatory effect of taxifolin and quercetin on oviposition by *C*. *maculata*. It was also found that quercetin had no negative effect on egg hatch rate [13]. 

Quercetin is a flavonol with the chemical formula of C_15_H_10_O_7_ and IUPAC name 2-(3,4-dihydroxyphenyl)-3,5,7-trihydroxychromen-4-one, and molecular weight of 302.238 g/mol (https://pubchem.ncbi.nlm.nih.gov). It has been discovered in numerous plant species and is one of several bioflavonoids responsible for the color of flower petals, attracting pollinating insects to flowers (and pollen), and some herbivorous insects to their host plants [14,15,16,17]. It is also known to stimulate oviposition in monarch butterflies [18]. Quercetin has repellent and insecticidal activity against some herbivorous insects, including aphids [19].

In this study, we further investigate the effects of quercetin on oviposition by *C*. *maculata*. We tested the hypothesis that quercetin stimulates oviposition by *C. maculata* in the presence of conspecifics and a tissue substrate. The key objectives were to evaluate the effects of quercetin on (1) oviposition behavior and (2) female positioning in cages in the presence or absence of a preferred oviposition substrate, i.e., tissue paper. 

## 2. Materials and Methods 

### 2.1. Insect Colonies and Food Sources

The *C*. *maculata* stock colony originated from adults provided by USDA colleagues in Beltsville, MD and Brookings, SD, USA. All of the life stages were reared separately in plastic containers in a climate room (24 °C, 50–60% RH, and 16 h photophase, year-round). Immatures were kept in Petri dishes (2.5 cm high, 9.0 cm diam). Adult mating pairs were kept in plastic cages (10 cm high, 8.0 cm diam) with screened lids and crumbled facial tissue paper (Kleenex^®^, Kimberly-Clark Corp., Neenah, WI, USA) as an oviposition substrate. Egg clutches were harvested from tissue paper for experiments. At the time that we commenced this study, we had maintained this stock colony for 33 consecutive generations without introducing any wild-type individuals from the field. Adults and larvae were fed an excess of frozen-fresh eggs of the Mediterranean flour moth, *Ephestia kuehniella* Zeller (Lepidoptera: Pyralidae), twice a week. A microcentrifuge tube containing distilled water (stoppered with a cotton wad) was present in Petri dishes at all times. Similarly, a cotton wad that was moistened with distilled water was provided in rearing cages at all times.

### 2.2. Quercetin Source

We purchased “synthetic” quercetin (greater than 98.0% pure powder) from MedChem Express LLC (Princeton, NJ, USA). We assumed that the behavioral and ovipositional responses of *C*. *maculata* would not differ between synthetic and natural sources of quercetin. 

### 2.3. Quercetin and Oviposition Behavior in Solitary Cages

**Figure 1:**
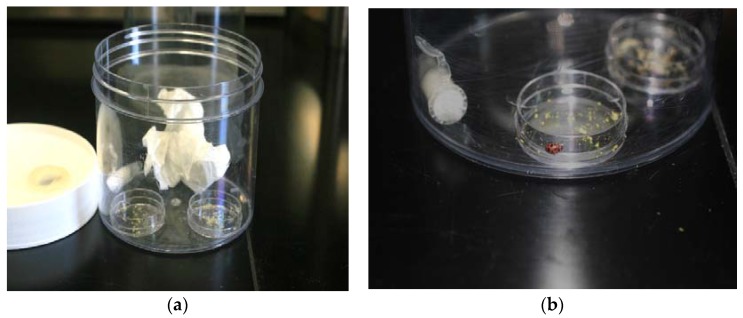
Image of 500 mL cage with chemical dish, food dish, water tube, and tissue substrate inside (**a**) and close-up of *C*. *maculata* adult clinging to the outer rim of chemical dish (**b**). Cage dimensions were 7 cm × 11 cm (height, diam). Chemical dish and food dish dimensions were the same, 1.0 cm × 3.5 cm (height, diam). Crumpled tissue paper occupied approximately 20% of the space in the cage.

Our three initial experiments tested the ovipositional responses of individual females to quercetin in 500 mL “solitary” cages (7 × 11 cm, height, diam; see Figure 1). Treatments included quercetin vs. none (experiment 1), quercetin vs. quercetin plus a tissue substrate (experiment 2), and quercetin plus a tissue substrate vs. a tissue substrate only (experiment 3). The tissue substrate was the same paper and from the same company, as used in our stock colony. Seven replicate cages were used for each treatment, for a total of 14 cages for each experiment. We measured oviposition site preference, egg clutch number per female, and eggs per clutch per female.

For each of the three experiments, we harvested approximately 100 one-month-old adults (combined sexes) from the same generation (33rd to 35th generations) from our stock colony, and then divided them into two plastic containers (9.5 cm high, 11 cm diam) for 2–3 days to ensure mating. Next, we isolated females in a Petri dish with a cotton wad moistened with distilled water, but no food, for 24 h in a growth chamber, to standardize hunger levels amongst females destined for bioassays. Hungry beetles are known to lay fewer eggs than well-fed ones [20]. 

Females were randomly selected from the plastic containers and then placed separately into the plastic solitary cages, one female per cage. All of the cages were provisioned with an excess of factitious food, i.e., *E*. *kuehniella* moth eggs in a tiny Petri dish (1.0 × 3.5 cm, height, diam) and distilled water in a microcentrifuge tube at the base of the cage. All of the cages contained another tiny Petri dish (a chemical dish, 1.0 × 3.5 cm) containing quercetin powder (1 mg) in the appropriate cage; the chemical dish was empty in cages not containing any quercetin.

Preliminary observations revealed that 1 mg of quercetin powder in the tiny dish were sufficient to illicit oviposition behavior of a single female. No other concentrations were used in these three experiments. Quercetin powder occasionally adhered to the body parts of females and their egg clutches in the dish. When this occurred, we added more quercetin to the chemical dish to ensure that approximately 1 mg was in the dish during the course of the experiments. 

Twice a day, once in the morning and again in the afternoon, we recorded the oviposition site preferences of females by noting the location of egg clutches inside the cages. Oviposition sites included the chemical dish, food dish, cage wall (lower wall, top wall), and tissue substrate (see experiment 3). We also counted the number of egg clutches per cage, then removed the egg clutches from the cages, and counted the number of eggs per clutch, using a stereo zoom microscope. We continued this procedure for 12 consecutive days for the three experiments.

The solitary cages were maintained in a growth chamber (24 °C, 60% RH, 16 h photophase), but removed twice a day to record oviposition data. Adult females were fed every other day, using the same food source (*E*. *kuehniella* eggs), as used in the stock colony. Old food and waste (fecal material) were discarded every other day.

### 2.4. Quercetin, Oviposition, and Female Locale in Communal Cages 

**Figure 2:**
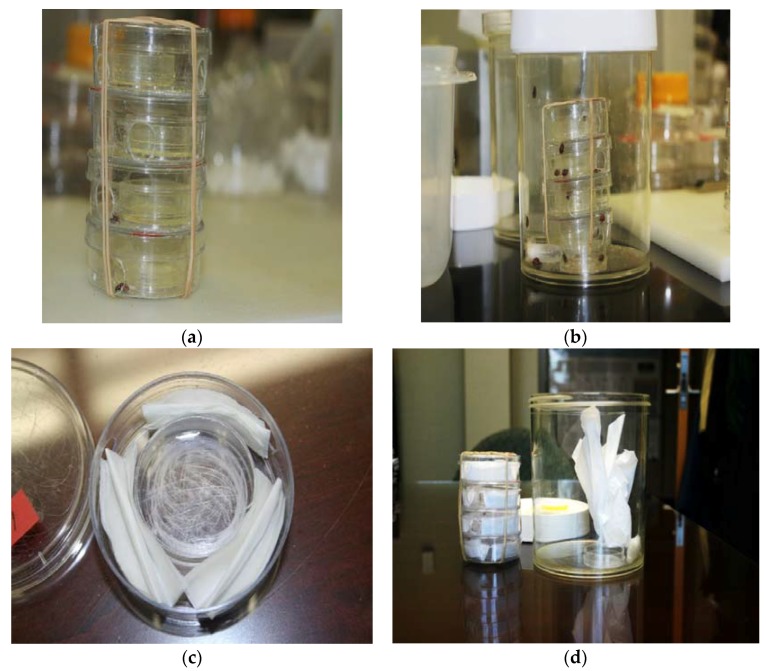
Image of oviposition chamber with chemical dishes containing quercetin powder (**a**), assembled 1 L cage with oviposition chamber, food dish, and water tube (**b**), oviposition unit with empty chemical dish (in center) with folded tissue paper (**c**), and disassembled 1 L cage showing the juxtaposition of lightly crumpled tissue substrate (20 cm × 20 cm sheet) outside the oviposition chamber (**d**). Oviposition chamber consisted of four, stacked units (2 cm high, 6 cm diam each), with a chemical dish (1 cm high, 3.5 cm diam) in the center. Three holes of the same diameter were bored in the walls of each unit to provide easy access to the chemical dish. Cage dimensions were 13 cm × 11 cm (height, diam). When folded, the tissue paper pieces inside a unit were 1.2 cm × 4.0 cm (height, diam).

Our final two experiments tested the ovipositional responses of grouped females to quercetin in 1 L “communal” cages (Figure 2). Cage dimensions were 13 cm × 11 cm (height, diam). A novel 4-unit oviposition chamber (Figure 2) was used in these two experiments, to reduce female-female bodily contact, restrict the chemical dishes (with or without quercetin) to one locality, and increase the amount of quercetin in cages, to accommodate grouped females. The oviposition chamber consisted of four, stacked units (2 cm high, 6 cm diam each), with a chemical dish (1 cm high, 3.5 cm diam) in the center. Three holes of the same diameter were bored in the walls of each unit in order to provide easy access to the chemical dish. The tissue substrate was the same paper (and from the same company) as used in our stock colony. When inside the oviposition chamber, the tissue was three equal-sized, folded pieces (1.2 cm high, 4.0 cm diam), encircling the chemical dish (Figure 2c). When outside the chamber, the lightly crumpled tissue substrate (20 cm × 20 cm sheet) occupied approximately 25% of the cage interior (Figure 2d).

In experiment 4, two replicate 1 L cages were used for both treatments, for a total of four cages and 40 females. In experiment 5, two replicate 1 L cages were used for each of the three treatments, for a total of six cages and 60 females. We measured the oviposition site preference, egg clutch number per female, and eggs per clutch per female. Ten females were randomly assigned to each treatment cage. Treatments included quercetin plus tissue substrate (outside the chamber) vs. tissue substrate only (outside the chamber) in experiment 4, and quercetin plus tissue (in the chamber) vs. quercetin plus tissue substrate (outside), and tissue substrate only (inside the chamber) in experiment 5.

As described in the previous section, 100 one-month-old adults (mixed sexes) of the same generation were harvested from the stock colony and established in large containers to ensure mating, before the mated females were separated in Petri dishes (with water only) for 24 h to standardize hunger levels. As mentioned in the previous section, females were randomly assigned to cages that were provisioned with an excess of factitious food (*E*. *kuehniella* eggs in a tiny Petri dish), and distilled water in a microcentrifuge tube at the base of the cage. In experiments 4 and 5, a 4-unit oviposition chamber was used (see Figure 2). Each chamber held a stack of four smaller Petri dishes, with each containing a tiny chemical dish. The four chemical dishes contained quercetin powder (2 mg) in the appropriate cage; the chemical dishes were empty in cages not containing any quercetin.

Based on preliminary assessments, the 2 mg of quercetin powder in the four stacked dishes was sufficient to elicit oviposition behavior of the 10 females that had easy access to the quercetin through entry/exit holes bored into the sides of each of the four units of the chamber. This was the only concentration used in these two experiments. 

Twice a day, once in the morning and again in the afternoon, we recorded the oviposition site preferences of females by noting the location of egg clutches inside the cages. Oviposition sites included the chemical dish, food dish, cage wall (lower and top wall combined), and tissue substrate. As in the previous section, we counted the number of clutches per cage, removed them from the cages, and counted the eggs per clutch, using a stereo zoom microscope. We continued this procedure for 12 consecutive days for these final two experiments.

As in the previous section, the cages were maintained in a growth chamber (24 °C, 60% RH, 16 h photophase), but removed twice a day to record oviposition data. The location of females in each cage was recorded twice a day, once in the morning (at approximately 09:00 h), and in the afternoon (at approximately 16:00 h). For the ease of recording, and reduce the disturbance to females while checking the locations of the 10 females in each treatment cage, we designated distinct locations. In experiment 4, distinct locations were (1) inside the chamber, (2) on tissue substrate, and (3) others, combined. In experiment 5, the distinct locations within the cage were (1) inside the chamber, and (2) outside the chamber.

Adult females were fed every other day, using *E*. *kuehniella* eggs, as used in the stock colony. Old food and waste (fecal material) were discarded every other day.

### 2.5. Statistical Analysis

Data were analyzed following a completely randomized design. A two-way analysis of variance (ANOVA), using the Standard Least Squares procedure with *F* statistic, tested the effects of treatment and clutch site preferences of females in solitary and communal cage bioassays. A Student’s *t*-test and 1-way ANOVA were used to test for significance of treatments on egg clutch number (and egg number per clutch), irrespective of clutch site. A two-way ANOVA was also used to test for significance of treatments on female location in communal cages. Treatment means were significantly different following the *t*-test or ANOVA, if *p* < 0.05. When necessary, a Tukey’s (Tukey-Kramer HSD) test was used as a multiple comparison procedure after the ANOVA. JMP^®^ 12.0.1 (2012, SAS Institute Inc., in Cary, NC, USA) and SigmaStat 3.0.1 interfaced through SigmaPlot 12 (Systat Software Inc., in Richmond, CA, USA) software assisted with the analysis of data. 

## 3. Results

### 3.1. Quercetin and Oviposition Behavior in Solitary Cages

#### 3.1.1. Experiment 1: Quercetin or None

**Figure 3:**
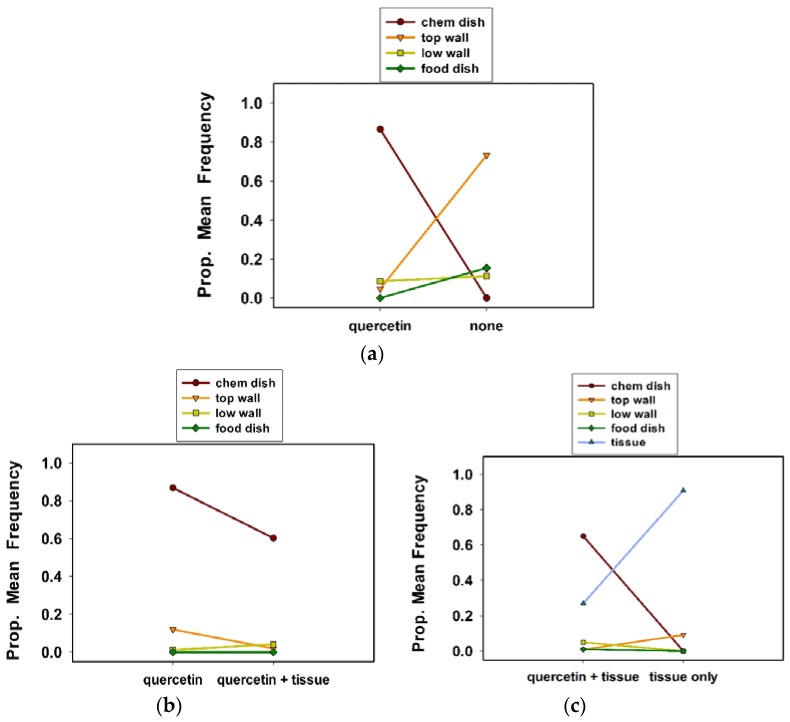
Proportional mean frequency of *C*. *maculata* egg clutches at sites in response to treatments in solitary cages in experiment 1 (**a**), experiment 2 (**b**), and experiment 3 (**c**).

Quercetin had significant effects on the selection of oviposition sites by *C*. *maculata* in solitary (500 mL) cages, i.e., housing a single female (Table 1, experiment 1; Tukey test values not shown). Females preferred to oviposit in the chemical dish in cages with quercetin versus an untreated check (Figure 3a). Proportionally, 0.87 of all clutches were in the chemical dish in cages with quercetin. In contrast, a proportion of 0.73 of all clutches were on the wall in cages without quercetin. Although of less importance, more clutches were in the food dish in cages without quercetin than with it.

Regardless of oviposition site, females laid more clutches in cages with quercetin than without it (Table 2, experiment 1). Slightly less than one clutch per female per day was found in cages with quercetin, when averaged over 12 days. In contrast, the number of eggs per clutch did not differ between the treatments.

#### 3.1.2. Experiment 2: Quercetin or Quercetin Plus Tissue Substrate

Females more often chose to oviposit in the chemical dish in cages with quercetin alone than with quercetin plus a tissue substrate (Table 1, experiment 2; Figure 3b; Tukey test values not shown). Some clutches were laid on tissue (data not included) in the quercetin plus tissue substrate cages. Proportionally, 0.82 of all clutches were in the chemical dish in the quercetin cages; 0.60 of all clutches were in the chemical dish in cages with quercetin plus tissue. 

Regardless of oviposition site, females laid more egg clutches per day in cages with quercetin plus a tissue substrate (Table 2, experiment 2). Slightly more than one clutch per female per day was found in the cages with quercetin plus tissue, averaged over 12 days. As in the previous experiment, the number of eggs per clutch was not significantly different between treatments (quercetin versus quercetin plus tissue). A similar number of eggs per clutch were evident (Table 2, experiment 2).

#### 3.1.3. Experiment 3: Quercetin Plus Tissue Substrate or Tissue Substrate Only

A tissue substrate in both treatments revealed that females preferred to lay their egg clutches in the chemical dish in the quercetin plus tissue cages, rather than tissue only cages (Table 1, experiment 3; Figure 3c; Tukey test values not shown). A proportion of 0.65 and 0.27 of all clutches were in the chemical dish and on tissue, respectively, in quercetin plus tissue cages. A proportion of 0.86 of all clutches were on the tissue substrate in the cages with tissue only (Figure 3c). Only a proportion of 0.09 of all clutches were on the top wall in the cages with the tissue substrate only.

Regardless of the oviposition site, egg clutch number did not differ between the treatments (Table 2, experiment 3). Approximately one clutch per female per day was found in the treatment cages. Also, egg number per clutch did not differ between treatments (Table 2, experiment 3). 

### 3.2. Quercetin on Oviposition and Female Locale in Communal Cages with an Oviposition Chamber

#### 3.2.1. Experiment 4: Quercetin Plus Tissue (Outside) or Tissue Only (Outside)

**Figure 4:**
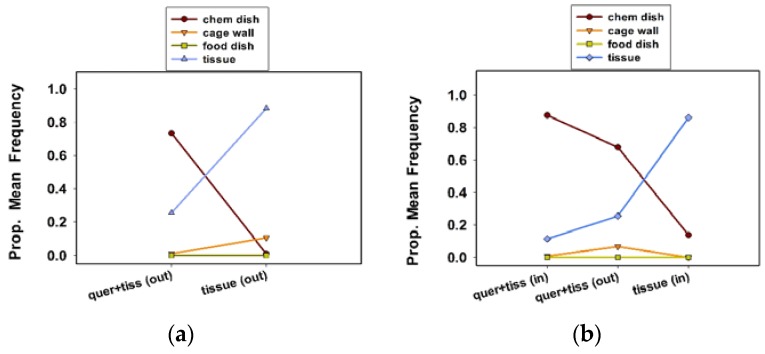
Proportional mean frequency of *C*. *maculata* egg clutches at sites in response to treatments in communal cages in experiment 4 (**a**), and experiment 5 (**b**).

In shared cages, i.e., housing 10 females, females preferred to oviposit in the chemical dish in cages with quercetin plus tissue (outside of chamber) than in the cages with tissue (outside of chamber) only (Table 3, experiment 4; Figure 4a; Tukey test values not shown). A proportion of 0.70 and 0.24 of all clutches were in the chemical dish and on the tissue, respectively, in the cages with quercetin plus tissue substrate. Note that a proportion of 0.88 and 0.11 of all clutches were on the tissue and the wall in cages with tissue only (Figure 4a).

Regardless of the oviposition site, clutch number did not differ between treatments (Table 4, experiment 4). The average clutch number was just 0.33–0.38 clutches per female per day. Also, egg number per clutch was unaffected by the treatments.

**Figure 5:**
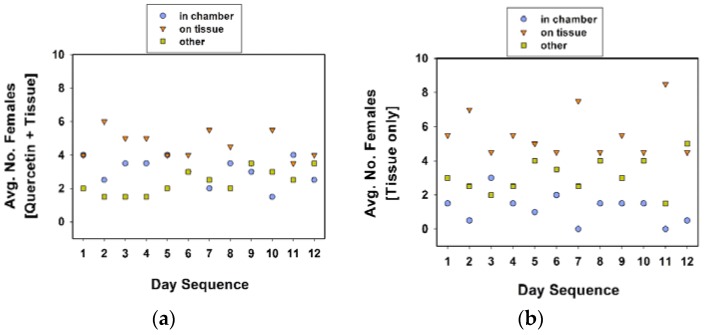
Average number of females in oviposition chamber, on tissue substrate, or other locales (i.e., cage walls, food dish). Treatments included quercetin powder in the chamber and tissue substrate outside (**a**), and tissue substrate outside the chamber (**b**). A total of 10 females (in each cage) were observed twice each day at approximately 09:00 and 16:00 h, over 12 days. Females were included in experiment 4.

When comparing the location of females in cages between treatment groups, more females were found inside the oviposition chamber in the quercetin plus tissue cages than the tissue only cages (Table 5, experiment 4; Figure 5; Tukey test values not given). In contrast, more females were on the tissue substrate in the tissue only cages than the quercetin plus tissue cages. Overall, females were more likely found on the tissue substrate (than in the oviposition chamber) in both treatments (Figure 5).

#### 3.2.2. Experiment 5: Quercetin Plus Tissue (Inside), Quercetin Plus Tissue (Outside) or Tissue Only (Inside)

More clutches were in the chemical dish in cages with quercetin plus tissue (in chamber) than quercetin plus tissue (outside chamber) or tissue (in chamber) only (Table 3, experiment 5; Figure 4b; Tukey test values not shown). A proportion of 0.88 and 0.68 of all the clutches were in the chemical dish in cages with quercetin plus tissue, inside and outside of the oviposition chamber, respectively. In cages with tissue (in chamber) only, a proportion of 0.86 of all clutches were on the tissue substrate.

Regardless of the oviposition site, clutch number was slightly greater in communal cages with quercetin plus tissue (in chamber) than quercetin plus tissue (outside) or tissue (inside) only (Table 4, experiment 5). Average clutch number was 0.52 clutches per female per day in the cages with quercetin plus tissue (in chamber). Notwithstanding, egg number per clutch was not affected by treatments (Table 4, experiment 5).

**Figure 6:**
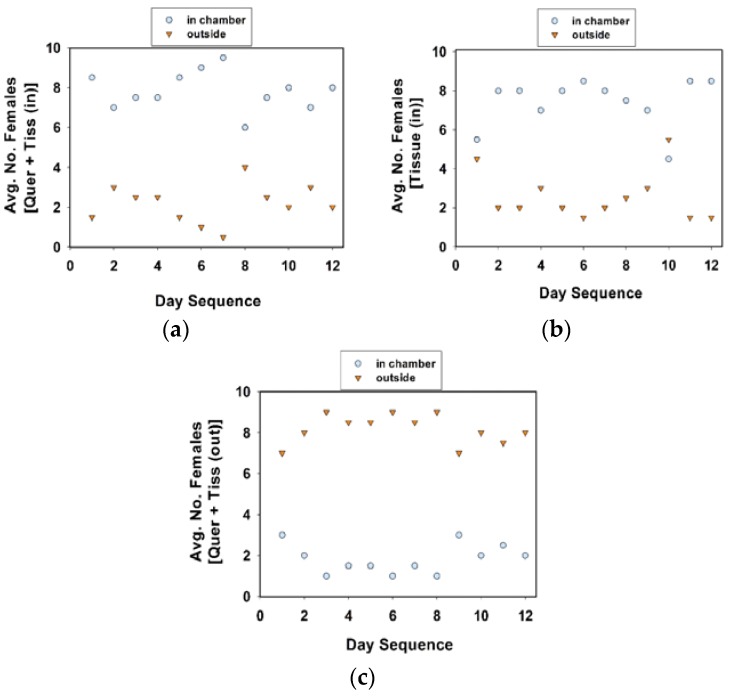
Average number of females inside or outside oviposition chamber. Treatments included quercetin powder and tissue substrate in chamber (**a**), tissue substrate only in chamber (**b**), and quercetin powder in chamber with tissue substrate outside (**c**). A total of 10 females (in each cage) were observed twice each day at approximately 09:00 and 16:00 h, over 12 days. Females were included in experiment 5.

A comparison of the location of females in communal cages revealed that fewer females were in the oviposition chamber in cages with quercetin plus tissue (outside) than in cages with quercetin plus tissue (inside) or tissue (inside) only (Table 5, experiment 5; Figure 6; Tukey test values not given). The number of females on the tissue substrate and other locations were combined. Females were attracted to the tissue substrate, whether it was inside or outside of the oviposition chamber, in cages with or without quercetin (Figure 6). 

## 4. Discussion

In cost-effective mass rearing systems, which are designed to increase the production of progeny for augmentative biological control, oftentimes natural prey are not used [1,2]. Aphids are the preferred prey for many ladybirds. But, aphids are difficult or impossible to rear in a cost-effective manner, because live plants (hosts) must be supplied as a food source. Production of plants for aphids is labor intensive and not practical for long-term production of ladybird beetles. Consequently, we have been experimenting with alternative food sources in lieu of aphids in our ladybird production system. A disadvantage of using non-aphid foods is that chemical cues that stimulate oviposition are often not present. As a result, chemical stimulants are necessary to boost the oviposition of ladybirds fed alternative foods or artificial diets. Natural compounds or molecules from a plant source rather than animal source (i.e., aphid tissues or aphid by-products, e.g., honeydew) would be ideal. This leads to our rationale for conducting this research. We have been testing various bioflavonoids as stimulants for oviposition in ladybirds. 

In a recent investigation, it was discovered for the first time that three bioflavonoids, quercetin, taxifolin, and naringenin (to a lesser extent), in bioassays in 250 mL cages, stimulated oviposition by *C*. *maculata* [13]. We have expanded our knowledge of the relationship between quercetin and this ladybird in this study. Our observation that females preferentially oviposited in a chemical dish containing quercetin, at the base of 500 mL solitary cages (housing one female), rather than on the cage walls or on tissue paper, confirms the attraction to and the stimulatory effect of this compound on females in a larger cage. Notably, in the presence of a tissue substrate, which occupied a much larger surface area than the chemical dish, females showed a preference for ovipositing in the chemical dish, suggesting that quercetin was more attractive than tissue paper. 

The observation that quercetin occasionally increased the number of egg clutches per female in 500 mL solitary cages was expected on the basis of previous research. For example, quercetin and naringenin stimulated slightly more egg clutches per female in 250 mL cages, than in the control cages—lacking any bioflavonoid [13]. The mechanism of action that triggers the physiological response in females to oviposit in close proximity to quercetin and to occasionally produce more egg clutches is unknown. Although not tested, doubling the cage size did not apparently affect the clutch number (0.82 clutches per female per day in 250 mL cage [13]; 0.80–0.92 clutches per female per day in 500 mL cage, in this study).

Quercetin does not harm *C*. *maculata* females. In fact, we have observed females “tasting” small amounts of quercetin powder, suggesting that taste chemoreceptors on the mouthparts recognize this compound as being suitable for consumption. Undigested fragments of quercetin powder have been found in their faeces (ZW, unpublished observations). The nutritional value of quercetin powder to female reproduction is unknown. Whether females must taste, touch, or digest quercetin (or any other bioflavonoids) to stimulate oviposition or boost egg production needs further study. Quercetin powder often adheres to the surface of freshly-laid *C*. *maculata* eggs, but it does not reduce the hatch rate [13]. Although not being tested in a rigorous experiment, hatching *C. maculata* first instars were not affected negatively when contacting quercetin powder.

Research on the effects of quercetin on larvae of an herbivorous ladybird *Epilachnia paenulata* (Germar), which feeds on curcubit leaves, proved that this compound had little or no effect of leaf consumption and the larval weight at concentrations from 0.1 to 50 μg/cm^2^ in laboratory bioassays [21]. Quercetin functions as a phagostimulant at low concentrations, but a feeding deterrent at high concentrations for *E*. *paenulata*. 

Although quercetin can incite females to increase clutch number, females never produced more than two clutches in a 24 h time frame. Quercetin never incited females to increase egg number per clutch in this study. In fact, neither quercetin nor taxifolin increased egg number per clutch in solitary cages in a previous investigation [13]. The simplest explanation for this observation is that females are limited in the number of eggs that they can produce over a given time period, because of the rate at which eggs mature in their ovaries. Other researchers have found that egg maturation rate was more dependent on a physiological response to food quality, ambient temperature, and ovariole size in ladybirds [22]. 

To facilitate cost-effective and space-efficient mass rearing, grouping ovipositing adults in the same cage would be ideal. Commercial scale rearing could involve placing hundreds of ladybird adults (males and females) in the same cage, provisioned with an oviposition stimulant (such as quercetin) and some type of oviposition substrate. Quercetin would encourage females to oviposit in oviposition chambers, placed in a restricted region in these cages, and to facilitate rapid harvesting of egg clutches each day and limiting egg cannibalism, by females and/or males. Thus, our attempt to foster a situation where females were restricted to ovipositing in the company of conspecifics, yielded interesting results. Our observation that females preferred to oviposit in the chemical dishes inside a novel oviposition chamber in 1 L communal cages containing quercetin powder, in the presence or absence of a tissue substrate, further confirms the capacity of this compound to stimulate oviposition. We used tissue paper as an oviposition substrate in our rearing system (stock colony) for this ladybird beetle, indicating that females had exposure to this substrate type prior to placement in experiments. Tissue paper, filter paper, and paper hand toweling are often deployed as oviposition substrates for aphidophagous ladybird beetles in the laboratory [6,7,23]. Nevertheless, quercetin appears to be a stronger oviposition stimulant than tissue paper, based on our cage bioassays.

The two- to three-fold decrease in egg clutch number per female per day in communal cages, in comparison to number per female per day in solitary cages (compare Table 2 and Table 4), suggests some female-female interference in the communal cages. Frequent bodily contact between females might discourage oviposition. Reluctance to oviposit in response to crowding of adults in a container, rather than lack of suitable prey (quality or quantity) has rarely been reported for adult ladybirds [6]. Adults of the ladybird *Propylea dissecta* (Mulsant) reduce their fecundity when female density (and the density of chemical tracks deposited by females) increases in small arenas, Petri dishes [24]. Perhaps, females decide to oviposit, or not, depending on a certain threshold concentration of chemical(s) released by conspecifics. Chemical cues could signal the threat of cannibalism. Nonetheless, we did not detect any cannibalism of eggs by adult females in the cages in our experiments.

The observation that the number of egg clutches per female was slightly greater in the communal cages with quercetin plus tissue (in the chamber) suggests that the presence of quercetin and tissue in close proximity occasionally triggers a stronger ovipositional response than either quercetin or tissue alone. We observed females “resting” on tissue whether it was inside or outside of the oviposition chamber, suggesting that they are attracted to the tissue because of the presence of chemical cues that are emanating from the paper or because the tissue provides a more secure grip for their tarsi than the plastic surfaces of the cage. The placement of tissue in close proximity to quercetin in rearing cages (in a mass rearing operation) could be the best strategy to stimulate more localized oviposition and perhaps more egg production by *C*. *maculata* females.

## 5. Conclusions

Quercetin stimulated oviposition by *C*. *maculata* in solitary (1 female) and communal (10 female) cages. In solitary cages devoid of quercetin, females preferred to oviposit on tissue. Occasionally, quercetin (and quercetin plus tissue) incited females to generate slightly more egg clutches, but not more eggs per clutch. In communal cages, females preferred to oviposit inside a novel oviposition chamber containing quercetin with or without tissue. Egg cannibalism by females was not detected in the oviposition chambers. Females often rested on tissue whether it was inside or outside of the oviposition chamber. This study provides evidence that quercetin can be utilized as an oviposition stimulant by *C*. *maculata* in rearing systems. Future research should determine whether this compound could stimulate oviposition in other ladybird species.

## Figures and Tables

**Table 1:** Two-way analysis of variance (ANOVA) statistics of the interaction between treatments and clutch site selection by *C*. *maculata* females in solitary ^1^ cages.

Experiment	Source of Variation	*F*	df	*p*
1	Treatment × Clutch Site	121.75	3, 88	<0.001
	Treatment	0.003	1, 88	0.95
	Clutch Site	41.72	3, 88	<0.0001
2	Treatment × Clutch Site	4.42	3, 88	0.006
	Treatment	7.41	1, 88	0.008
	Clutch Site	166.94	3, 88	<0.001
3	Treatment × Clutch Site	53.60	4, 110	<0.0001
	Treatment	0.123	1, 110	0.73
	Clutch Site	66.74	4, 110	<0.0001

^1^ One female was inside each replicate 500 mL cage. Refer to Figure 3 for graphical displays of clutch site preferences. Sample size was 96, 96, and 120 for experiments 1, 2, and 3, respectively. *p* < 0.05 indicates significant differences amongst sources of variation.

**Table 2:** Mean (±SE) number of egg clutches per *C*. *maculata* female per day and mean (±SE) number of eggs per clutch, regardless of oviposition site, in solitary ^1^ cages.

Experiment	Treatments	Number of Egg Clutches	Number of Eggs per Clutch
1	Quercetin	0.80 ± 0.08	11.36 ± 0.64
	None	0.59 ± 0.04	11.67 ± 0.89
		*t* = 2.38 df = 22 *p* = 0.026	*t* = 0.28 df = 22 *p* = 0.78
2	Quercetin	0.92 ± 0.05	11.63 ± 0.60
	Quercetin + Tissue	1.15 ± 0.06	10.99 ± 0.55
		*t* = 2.96 df = 22 *p* = 0.007	*t* = 0.78 df = 22 *p* = 0.44
3	Tissue	0.92 ± 0.02	12.94 ± 0.61
	Quercetin + Tissue	0.94 ± 0.07	12.20 ± 0.59
		*t* = 0.31 df = 22 *p* = 0.76	*t* = 0.86 df = 22 *p* = 0.40

^1^ One female was inside each replicate 500 mL cage for 12 days. Data were pooled across replicate cages and averaged for 12 days per treatment group. Sample size was 24 for all experiments. A Student’s *t*-test was used for data analysis in experiments.

**Table 3:** Two-way analysis of variance (ANOVA) statistics of the interaction between treatments and clutch site selection by *C*. *maculata* females in communal ^1^ cages.

Experiment	Source of Variation	*F*	df	*p*
4	Treatment × Clutch Site	688.43	3, 8	<0.001
	Treatment	1.00	1, 8	0.34
	Clutch Site	642.54	3, 8	<0.001
5	Treatment × Clutch Site	82.41	6, 12	<0.001
	Treatment	0.073	2, 12	0.93
	Clutch Site	197.99	3, 12	<0.001

^1^ Ten females were in each of 2 replicate 1 L cages for 12 days. Refer to Figure 4 for the illustration of oviposition site preferences. Sample size was 16 and 24 for experiments 4 and 5, respectively. *p* < 0.05 indicates significant differences amongst sources of variation.

**Table 4:** Mean (±SE) number of egg clutches per *C*. *maculata* female per day and mean (±SE) number of eggs per clutch, regardless of site selection in communal ^1^ cages.

Experiment	Treatments	Number of Egg Clutches	Number of Eggs per Clutch
4	Quercetin + Tissue (out)	0.34 ± 0.05	12.41 ± 0.85
	Tissue (out)	0.38 ± 0.03	14.40 ± 0.91
		*t* = 0.74 df = 46 *p* = 0.46	*t* = 1.6 df = 46 *p* = 0.12
5	Quercetin + Tissue (in)	0.52 ± 0.03 b	10.55 ± 0.39 a
	Tissue (in)	0.42 ± 0.03 a	11.60 ± 0.42 a
	Quercetin + Tissue (out)	0.39 ± 0.03 a	11.03 ± 0.51 a
		*F* = 5.78 df = 2, 69 *p* = 0.005	*F* = 1.40 df = 2, 69 *p* = 0.25

^1^ Ten females were in each of two replicate 1 L cages for 12 days. Sample size was 48 and 72 for experiments 4 and 5, respectively. A Student’s *t*-test and an *F*-test (one-way ANOVA) were used for data analysis for experiments 4 and 5, respectively. The same alphabet after the mean value in a column indicates no significant difference (*p* < 0.05) between treatments.

**Table 5:** Two-way analysis of variance (ANOVA) statistics of the interaction between treatment and location of *C*. *maculata* females in communal ^1^ cages.

Experiment	Source of Variation	*F*	df	*p*
4	Treatment × Female Locale	24.99	2, 138	<0.001
	Treatment	0.54	1, 138	0.46
	Female Locale	58.98	2, 138	<0.001
5	Treatment × Female Locale	531.08	2, 138	<0.001
	Treatment	0.013	2, 138	0.99
	Female Locale	33.07	1, 138	<0.001

^1^ Ten females were inside each of two replicate 1 L cages for 12 days. Refer to Figure 5 and Figure 6 for graphical displays of female location pooled across cages and averaged per day. Sample size was 144 for experiments 4 and 5. *p* < 0.05 indicates significant differences amongst sources of variation.
